# Immigration and Integration Policy and Labour Market Attainment Among Immigrants to Scandinavia

**DOI:** 10.1007/s10680-018-9483-3

**Published:** 2018-03-21

**Authors:** Vibeke Jakobsen, Tomas Korpi, Thomas Lorentzen

**Affiliations:** 1 Danish Center of Social Science Research, Copenhagen, Denmark; 2 Stockholm University, Stockholm, Sweden; 3 University of Bergen, Bergen, Norway

**Keywords:** Immigration policy, Integration policy, Employment, Earnings, Benefits, Training programmes

## Abstract

Insufficient integration of immigrants into the labour market has been identified as a major problem in the Scandinavian countries Denmark, Norway and Sweden. Integration depends, inter alia, on immigration and integration policy, and for most of the post-war period the policies of the three countries displayed strong similarities. However, in the early 2000s Denmark increasingly deviated from its two neighbours, introducing more restrictive immigration and stricter integration policies. Comparing both pre- and post-reform immigrants across Scandinavia, we assess the wider impact of this comprehensive policy reversal by tracking the evolution of employment and earnings gaps between 1993 and 2006. We use large data sets with individual-level register information allowing us to account for immigrant labour force composition and to examine sub-groups of immigrants. The results do not indicate that the Danish reforms had any clear-cut effect on either employment or earnings among non-Western immigrants. Moreover, integration in Norway and Sweden was not unequivocally worse despite the absence of similar reforms, raising questions regarding the aptness of the Danish reversal.

## Introduction

Recent decades have witnessed dramatic changes in European migration patterns (see e.g. Castles et al. 2014, or Van Mol and de Valk 2016, for overviews). In the 1970s, the labour migration to Northern and Western Europe from Southern Europe (and former colonies) that characterised the early post-WWII period ended. In Northern and Western Europe, it was replaced by family and refugee migration, whereas Southern Europe now saw a rise in work-related (and partly irregular) immigration. In the 1990s and 2000s, the introduction of the European Single Market and the expansion of the European Union (EU) renewed labour migration to Northern and Western Europe, supplementing the humanitarian migration. These EU level changes also transformed eastern European countries into sending countries and accentuated southern European countries’ position as receiving countries. However, the financial crisis of 2008 revived labour migration from Southern Europe to Northern and Western Europe. Finally, around 2015 many European countries saw a rise in asylum applications, some drastically so (Hatton 2017).

These developments were partially driven by (EU or national) changes in immigration policies, for instance with regard to work permits and family reunification. However, after 1990 concerns regarding low employment and high rates of welfare recipiency prompted some countries to reform their labour market-related integration policies, i.e. the programmes and benefits available to recent immigrants believed to impact their integration on the labour market. This often involved expanding training and employment programmes, and/or reducing out-of-work benefits. Integration reforms were occasionally supplemented with reforms to immigration policies, generally involving tightened eligibility criteria (Eurofound 2016; Geddes and Scholten 2016).

This broad-brush picture highlights the regional differences within Europe, contrasting the north and the west with the east and the south. The Scandinavian countries Denmark, Norway and Sweden can in many ways be seen as typical of North-Western Europe, with migration patterns very similar to those outlined above.

The three countries are also countries where it is relatively difficult for immigrants to enter the labour market. An analysis from the OECD covering the period 1995–2005, for instance, showed the Scandinavian countries to consistently be among the worst performers in terms of native-immigrant participation, unemployment and employment gaps (OECD 2007). Consequently, immigrants to Scandinavia also had on average lower incomes and higher poverty rates and were, relative to the majority populations in the three countries, overrepresented among social assistance recipients (Galloway et al. 2015).

Immigrant success in the labour market depends on factors related to the immigrants themselves, to the country of origin and to the country of destination. Focusing on the latter, we here find several potential explanations for the poor integration of immigrants in Scandinavia, e.g. languages often unknown to immigrants, high minimum wages and labour demand emphasising high levels of formal qualifications. The potential explanations also include the countries’ immigration and integration policies. The policies in the Scandinavian countries have here traditionally been quite similar, with substantial policy feedback occurring between the countries. However, in the 2000s Denmark distanced itself from Norway and, especially, Sweden by introducing more restrictive immigration and stricter labour market integration policies. Two examples of the Danish makeover are the reforms in 2002 when Denmark tightened immigration policy disallowing family reunification for spouses below 24 years of age, and also changed integration policy introducing reduced social assistance benefits for immigrants. This has been described as Denmark emphasising the stick, Sweden the carrot and Norway a combination of the two (Brochmann and Hagelund 2012).

The purpose of this paper is to examine the consequences of this Scandinavian policy divergence for immigrant integration. Although there are studies of separate reforms in the different countries (discussed further below), we here attempt to make an overall assessment of the impact of the countries’ different policy choices by studying multiple outcomes and by placing these choices in relation to each other. Examining specific impacts of separate reforms is of obvious importance, yet aggregate appraisals of broad policy changes are also needed. Policy reform is often comprised of many distinct components and can therefore be regarded as packages wherein the separate elements may have both reinforcing and counteracting effects. An aggregate appraisal can then provide information on the overall effect. A general assessment is furthermore necessary in relation to public debate, as policy makers often, for instance in the on-going European policy debates, present policies to the electorate as packages. A party platform may thus be presented as being “tough” or “generous”, rather than in terms of the details of specific proposals.

One of the stated aims of the Danish policy reconfiguration was to increase employment among immigrants (Tranæs 2014), and, to the extent the shift in policy was successful, we would expect immigrants arriving after the reforms to have higher employment rates than those coming earlier. Likewise, post-reform immigrants should have higher employment rates relative to the majority populations in the Danish than in the Norwegian and Swedish labour markets. However, the integration policy reforms aimed to achieve this in part by making immigrants accept less attractive jobs, so a potential effect of the policies may also be lower relative earnings among post-reform immigrants in Denmark. Against this background, we analyse the development of relative employment and earnings comparing pre- and post-reform immigrants in Scandinavia. We use individual-level register data from 1993 to 2006, a period spanning the Danish reforms, and focus on natives and non-Western immigrants of working age (30–59).

## Immigration and Integration Policies in Scandinavia

The post-war migration histories of Denmark, Norway and Sweden are in many respects very similar, and also similar to those of the other north-western European countries described above. During the period of labour recruitment, immigrant workers in Sweden came primarily from Finland, Italy, the former Yugoslavia, Greece and Turkey; in Denmark they came mainly from the former Yugoslavia, Turkey, Pakistan and Morocco, while immigrants to Norway came chiefly from Pakistan, Turkey and Morocco. In Scandinavia, this period ended in 1975 when Norway became the last country to terminate recruitment (Brochmann and Hagelund 2011). Immigration during subsequent decades was instead characterised by family reunification and asylum (Berge et al. 2009; Djuve and Kavli 2007; Dølvik and Friberg 2008). The number of refugees, primarily originating from the former Yugoslavia, Afghanistan, Iraq and Somalia (Nordic Statistical Yearbook 2012), increased significantly in all three countries in the 1980s and, in particular, in the 1990s (Berge et al. 2009).

Although there were differences with regard to the precise timing of the changes in policy, up until the early 2000s the evolution of immigration policy in the three countries may nonetheless be described as roughly comparable. At this point, Denmark began to move away from Norway and Sweden, tightening rules on family reunification and asylum (Berge et al. 2009). The most important regulatory changes were introduced in July 2002 and included the introduction of the 24-year requirement (no family reunification for spouses younger than 24 years), the attachment requirement (the combined attachment of a couple to Denmark must exceed their attachment to other countries) and the support requirement (the Danish resident must be able to provide for arriving family members). This tightening of rules resulted in a drastic decrease in number of marriage migrants coming to Denmark (Andersen 2007; Schmidt et al. 2009) and a decrease in the number of refugees (Nordic Statistical Yearbook 2012). In contrast, no similar restrictions on immigration were introduced in Norway or Sweden at this point, and the structure of immigration therefore changed less than in Denmark.^1^

The countries also diverged with regard to their treatment of migrants from the new European Union (EU) member states following EU enlargement in May 2004 (Dølvik and Friberg 2008). Denmark and Norway both requested a transition period lasting until 2009 before mobility became completely unregulated, something Sweden did not. Nonetheless, the strong Norwegian labour market meant that Norway received more than twice the number of EU migrants than Denmark and Sweden together (Berge et al. 2009). In all three countries a large number of immigrants arrived from Poland, but many also came from countries such as Lithuania and Rumania (Nordic Statistical Yearbook 2012).

The integration policies of the three countries also show many similarities up until the late 1990s—but some clear differences afterwards. During the 1980s and 1990s, it gradually became clear that the labour market position of immigrants had deteriorated dramatically. In all three countries, low employment rates together with high rates of social assistance recipiency led to debates around welfare rights and work incentives, and to reforms of benefits systems and the introduction of measures targeting immigrants (Brochmann and Hagelund 2010). The reforms may be placed under three broad headings: changes in benefits, in training programmes and other reforms potentially affecting the labour market position of immigrants.

Regarding benefits, Denmark was the only Scandinavian country to introduce cuts in benefits targeted at immigrants (Brochmann and Hagelund 2010; Djuve and Kavli 2007). This was initiated in January 1999 when the Integration Act established an obligatory Introduction Programme providing all new non-EU immigrants with an allowance substantially below the social assistance benefits previously offered. The benefit reduction was short-lived, although the programme continued benefits were restored after only 1 year. However, in July 2002 the Start Help benefit was introduced. Start Help encompassed people who had resided abroad for at least seven of the most recent 8 years and included an allowance set 35% below the social assistance benefit to which these immigrants previously would have been eligible (Pedersen 2013). In contrast, although similar programmes existed in both Norway and Sweden (programmes established in 2003 and 1994, respectively), the allowance did here not differ significantly from standard social assistance.

Another reform to Danish social assistance was the introduction of a maximum for the total amount of social assistance benefits, housing subsidies and the so-called specific support recipients could obtain after an initial six-month period. Recipients were first affected by this ceiling in January 2004. In addition, married couples on social assistance experienced a cut in benefits (of approximately 500 DKK for each spouse) after 6 months of benefit receipt. None of the programmes was explicitly targeted at newly arrived immigrants or even at immigrants in general, yet a large majority of those affected were immigrants and newly arrived immigrants disproportionally so (Brochmann and Hagelund 2011; Graversen and Tinggaard 2005; Pedersen 2013). As noted, no similar benefit reductions were introduced in Norway and Sweden.^2^

The contents of the Introduction Programme and the corresponding programmes in Norway and Sweden were very similar; language courses and work-oriented training typically involving 2–3 years of full-time participation. Despite these similarities, there were, in addition to the differences in allowances, potentially important differences in the regulation of the programmes. The programmes were for instance mandatory for immigrants in Denmark and Norway, while participation was voluntary in Sweden. Furthermore, central government exerted less control over the programmes in Sweden than in Norway and, in particular, Denmark. In the latter two countries, municipalities were obliged to set up introduction programmes, while this was voluntary in Sweden. Furthermore, integration law in Denmark regulated the contents of the programme in detail, whereas Swedish regulations included very few explicit guidelines. Norway was located somewhere in between, containing fairly general specifications regarding content leaving substantial leeway for municipal variation (Berge et al. 2009; Brochmann and Hagelund 2010; Djuve and Kavli 2007). It may also be noted that financial reimbursement for the programmes varied, with Danish municipalities being fully compensated, Norwegian ones compensated through a block grant and Swedish municipalities only being compensated to a relatively limited extent (Djuve and Kavli 2007).

## Theory and Earlier Empirical Evidence

Our theoretical starting point for these analyses are economic theories of migration and job search, theories often referred to in discussions around immigration and labour market-related integration policies. Economic migration theory generally assumes that migration is the outcome of differences in earnings opportunities in the countries of origin and destination, and that the magnitude and characteristics of migration are affected by the relative levels and distribution of skills in the two countries, the transferability of skills, and the costs of migration (see e.g. Bodvarsson and van den Berg 2013, for a review). Immigration policies in the destination countries may affect both the number and characteristics of immigrants. Quota systems will likely impact primarily the number of migrants, while skill-based immigration systems will affect their characteristics (e.g. Clark et al. 2007). Integration policies may also impact the size and selection of immigration. Generous benefits could, for instance, increase both the number of migrants in general as well as the number of those most likely to receive the benefits (Borjas 1999).

The basic assumptions of job search theory are similar, although here the focus lies more on the process through which information about different alternatives is collected and on how they are evaluated. In the basic model, the decision regarding whether to accept a job offer is dependent on the frequency with which jobs are found (the arrival rate), and on whether the job exceeds one’s minimum requirements regarding wages and other working conditions (the so-called reservation wage). The reservation wage will in turn depend on the other available jobs as well as on the alternatives if one is not employed, in particular any benefits available to those not working. In the basic model, benefits will make the job seeker more selective about which jobs to accept, lowering the likelihood of finding an acceptable job but at the same time raising the expected wage if an acceptable one is found (see e.g. Rogerson et al. 2005, for a review).

What does this brief theoretical review suggest regarding the consequences of the changes in policies? On likely consequence is that the stricter immigration policies introduced in Denmark should generate a successive transformation of the immigrant labour force. The regulatory changes should reduce the proportion of newly arrived immigrants (in particular of refugees) and increase the proportion of arrivals coming to work and study (Tranæs 2014).

While the economic theories of migration suggest that the tightening of Danish immigration regulations could be expected to impact immigration, they offer little theoretical guidance on the impact of immigration reform for immigrants’ employment and earnings prospects. However, from a search-theoretical perspective, the compositional change towards more work-related immigration could potentially lead to an increase in, for example, the average arrival rate, and the reforms could consequently be expected to increase the immigrant employment rate. These changes to immigration policy would seem unlikely to lead to any changes in wages, so changes in earnings inequality would if anything involve a decreased earnings gap as the labour market integration of immigrants improves.

As for the reforms to integration policy, it could again be conjectured that the benefit reductions may have increased employment among immigrants in Denmark. The migration models outlined above would for instance predict that lower benefits would not only lower immigration, but also shift the composition of immigration towards individuals more likely to find employment. Increased employment would also be predicted in a standard job search model: a reduction in benefits leads to lower reservation wages and/or higher arrival rates (e.g. through intensified job search) both of which raising the transition rate into employment. A search model, however, also predicts that lowering benefits may increase the rate of exit out of the labour force (Tatsiramos and van Ours 2014).

As noted, the programmes not only entail the receipt of benefits, there is also a training component. Again referring to job search theory, this could be expected to improve the job arrival rate and/or the wage offer distribution, both in turn improving employment possibilities (Van den Berg et al. 2009). The content of the programmes generally appears very similar across the three countries and would be expected to strengthen the labour market position of immigrants. However, the central control of the content of the training programmes as well as the full compensation received by the municipalities suggests that the Danish introduction programmes might be of better quality than their Norwegian and Swedish counterparts. Coupled with mandatory participation, this implies that the Danish programmes would strengthen the position of immigrants more than the corresponding programmes in Norway and Sweden.^3^

Taken together, the reforms to Danish labour market integration policy could be hypothesised to increase employment among immigrants in Denmark relative to those in Norway and Sweden. To the extent that this occurs, it could be the outcome of a higher arrival rate, better job offers, or lower reservation wages. The implications of the two latter factors for wages are obviously contradictory: the improved offers suggesting higher and the reduced aspirations lower wages. This would in turn imply that immigrants could end up in well-paid and secure or poorly paid and insecure jobs. In the first case, we would have an increase in the relative earnings of employed immigrants, in the latter a decrease.

Evaluations of the Danish reforms have produced rather mixed results. The introduction of Start Help was, on the one hand, found to increase transitions into employment during the first 2 years following the reform, but also to increase the transition rate out of the labour force (Clausen et al. 2009; Huynh et al. 2010; Rosholm and Vejlin 2010). In this context, it is also interesting to note that positive employment effects were also found for the Norwegian integration programme (Kvinge and Djuve 2006), as well as for the very similar programme introduced in 1999 in neighbouring Finland (Sarvimäki and Hämäläinen 2016). Partly contradicting the above Danish results, although the introduction of the social assistance ceiling was found to decrease the benefits available to the affected families, no effect on job search or job finding were evident in the first 9–10 months after the reform (Graversen and Tinggaard 2005).

These evaluations provide very valuable information regarding particular aspects of the turnaround of Danish immigration and labour market integration policy. However, due to their focus on specific reforms, and furthermore only reforms to integration policy, they fail to examine the full implications of the transformation of Danish policy. Additionally, they provide no information regarding potential alternative policy choices.

The purpose of this paper is to provide such a broad assessment, examining not only employment but also earnings among pre- and post-reform immigrants to Denmark and using Denmark’s Scandinavian “twins” Norway and Sweden as yardsticks when judging the success of the Danish policy reversal. Although there are undeniable differences between the countries, we believe them sufficiently similar to provide an interesting comparison.

## Data, Measures and Analytical Strategy

### Data

The analyses are based on large administrative datasets covering roughly a 15-year period, from 1993 to 2006. More specifically, the data from Denmark cover 10% of native Danes and the total population of immigrants and descendants of immigrants, while the data from Norway and Sweden include the total population. We restrict the analyses to immigrants and natives of working age, more precisely those aged 30–59. By excluding individuals below the age of 30 or above the age of 59, we minimise the number of retirees and full-time students.

In all three countries, the data consist of information on annual income (including income from work as well as from other sources), on receipt of various kinds of transfers such as sick pay and unemployment compensation, as well as on demographic characteristics, education, place of residence and sector of employment.

Working with register data of this kind has some definite advantages. First, given the relatively small number of immigrants of working age, large sample size is particularly important in the analysis of immigrant integration on the labour market. This also allows us to examine sub-groups of immigrants such as those coming from a particular country/region. Second, the register data contain information on various types of income from the tax authorities that obtain earnings data directly from employers and data on transfers directly from the responsible agencies. This thereby avoids problems frequently associated with self-reported income, for instance recall error.^4^

### Measures

We define immigrants as persons born abroad, a definition that is advantageous in comparative analyses as it avoids the impact that differences in naturalisation rules may have for a citizenship-based definition. This implies that children born in the country of destination to persons born abroad, so-called second-generation immigrants, are not included among immigrants. Although this is a group of obvious interest, it is less relevant here as the policy reforms targeted newly arrived immigrants.

The Danish reforms presented above were introduced following debates centred on immigration from what we will here refer to as non-Western countries, i.e. from countries outside of Western Europe, USA, Canada, Australia and New Zealand; and similar debates also took place in Norway and Sweden. For this reason, our main analyses focus on the labour market integration of non-Western immigrants. However, we also conduct separate analyses of immigrants from the Horn of Africa (Djibouti, Eritrea, Ethiopia, Somalia and Sudan), Poland and Turkey. These are all relatively common countries of origin, and, as noted in the previous section, they also represent different waves of immigration to Scandinavia. This will thus give an indication of similarities and differences in labour market integration across quite diverse immigrant groups and also indicate the robustness of our conclusions regarding the group of non-Western immigrants as a whole. Finally, we also carry out separate analyses for men and women. Although we will be unable to present all our results due to space limitations (full details are, however, available upon request), we will make note of relevant comparisons as needed.

The earnings measure we use refers to individual annual earnings from paid work and income from self-employment. In other words, income from capital or from transfers of any kind is not included. Negative earnings are coded as missing, and earnings are top coded at 10,000,000 (Danish/Norwegian/Swedish) Crowns.

While detailed earnings data is an obvious advantage associated with the use of register data, information on other aspects of employment is often more problematic, less precise and not necessarily comparable. In our analyses of employment patterns, we therefore emulate, using the Social Exclusion and Labour Market Attachment (SELMA) model (Bäckman et al. 2011), the employment status classifications generated from labour force surveys.

The starting point for the model is the definition of a benchmark income used to classify income levels. This benchmark, the Nordic Base Amount (NBA), is set to 25 per cent of the median gross annual income in each country and year. The income limit distinguishing those able to support themselves through earnings, the so-called Core Labour Force, is then defined as annual earnings from work or self-employment of at least 3.5 NBA. This is roughly equal to the annual labour earnings obtained from full-time continuous employment at the minimum wages established in collective bargaining agreements in, for instance, the hotel and restaurant sector.

The employed are defined as those belonging to either the Core or the Unstable Labour Force, with the latter consisting of those with earnings between 0.5 and 3.5 NBA and no substantial income from public transfers such as sick pay, early retirement, unemployment compensation, or study allowance. Those with very low earnings and no transfer income or with non-negligible earnings in combination with significant transfer income (e.g. study allowances, unemployment benefits, or sick pay) are classified as non-employed.

### Analytical Strategy

We examine crude differences in employment and earnings between immigrants and natives as well as differences in predicted native-immigrant employment and earnings gaps. For the predictions, we estimate regression models of the general form

$$Y_{it} = {\text{X}}_{it}\upbeta + \varepsilon_{it}$$

where *Y* is a dependent variable differing across the analyses, X a vector of independent variables (largely) identical across analyses, β a corresponding vector of parameters to be estimated (including a constant), *ε* an error term, and *i* and *t* denote individual and time.

In our analyses of employment, the dependent variable is dichotomous equalling one if employed and zero otherwise. We here make use of probit models, and *Y* can in this context be thought of as an indicator of whether an underlying continuous latent variable Y* is positive or not (a case obtained if *ε* ~ *N*(0,1)). In our analyses of earnings, we instead have a continuous dependent variable where we are interested in the lower parts of the distribution. We therefore employ quantile regression, where *Y* is one of three separate percentiles of the earnings distribution, namely the 10th, 25th and 50th percentiles.

These models are estimated separately for natives and for immigrants in each country and each year. The variables included are sex, age (three age groups), education (five levels), a zero–one indicator for any children below age 18, and the municipal unemployment rate. In the analyses of earnings, we also include sector of employment (ten sectors). Finally, for immigrants we include years since migration (seven categories).

The results from the regression models are then used to predict earnings as well as the likelihood of employment for a 30–39-year-old male with compulsory education, at least one child, living in a municipality with 5% unemployment, and, in the case of earnings, working in the sectors wholesale and retail trade or hotels and restaurants. Among immigrants we make separate predictions for those who have been in the country less than 1 year, those with at least one but less than 2 years of residency, and those with at least two but less than 4 years of residency. Finally, the predictions for natives and for immigrants are used to calculate annual country-specific native-immigrant gaps in predicted employment and earnings, respectively.

Predicted gaps are of obvious interest here as they inform us about the gaps after accounting for compositional differences between the groups and the countries. Predicted gaps also allow us to tackle differences in macroeconomic conditions between the countries and over time. Immigrant employment is typically more affected by business cycles than native employment (see e.g. Dustmann et al. 2010), something which may impact the employment and earnings gaps. Although the evolution of the business cycle is largely comparable in the three countries, the early 1990s recession persisted longer in Sweden than in Denmark and Norway. Up until 1997, growth rates in Sweden were thus generally lower, particularly relative to Norway, but this changed and Sweden thereafter experienced stronger growth than its two neighbours. These cyclical differences are also reflected in, for example, aggregate employment rates. OECD data for the age group 15 to 64 (i.e. a broader age range than used in this paper) show small initial differences in employment levels, somewhat larger variation between 1995 and 1998 and thereafter differences similar to the original ones. We handle this variation by calculating predicted employment and earnings at the same level of unemployment (5%) in all three countries.

These analyses allow us to conduct multiple comparisons of immigrant integration, both within and across countries of destination and also across countries of origin. One such comparison involves different cohorts of immigrants within the three Scandinavian countries. In particular, the rapid succession of reforms in Denmark means that different immigrant cohorts were affected by the separate reforms to varying extent, and to capture such differences we distinguish immigrants based on their year of immigration. In our main analyses, we report results for immigrants with less than 1 year and with at least one but less than 2 years since migration, yet we have also analysed immigrants with at least two but less than 4 years since migration.

These comparisons amount to a difference-in-difference (DiD) analysis, where the basic idea is to examine changes in pre- and post-test measurements for treatment and control groups taking the between-group difference in the change over time as the treatment effect. We thus distinguish between treatment and control groups within Denmark based on years since migration, and it is important to note that the year of arrival of a particular years-since-migration category changes each year. This modelling strategy is the result of our desire to examine the consequences of a series of reforms introduced over a number of years, rather than a single reform introduced at one point in time.^5^

Close attention must therefore be paid to which years-since-migration category is affected by which reform in which year. Immigrants arriving in 1999 participated in the first version of the Danish integration programme, whereas prior arrivals did not. In other words, all immigrants with less than 1 year of residency in 1999 had taken part in the programme, while immigrants with more than 1 year since migration had not. Subsequent arrivals also took part in the programme, yet received the more generous benefit. In 2002, a minority of the immigrants with less than 1 year since migration arrived under the new immigration rules and instead received Start Help. In 2003, almost all immigrants with less than 1 year of residency had entered under the new family unification rules and received Start Help, whereas a minority of those with at least 1 year since migration and none of the earlier arrivals did. In 2004, all immigrants with zero or 1 year of residency had arrived under the new rules and received Start Help, and all immigrants, regardless of date of arrival, were also liable to face benefit reductions due to the benefit ceiling. (A small group of the most recent immigrants were also affected by the EU-enlargement restrictions.) In contrast, by 2005 almost all of the most recent immigrants were affected by all the new regulations, and in 2006 this also applied to the majority with 2–4 years of residency. We thus have a situation in which a series of reforms impact increasingly large proportions of recently arrived immigrants, with the key breaks being between 2002-2003 and 2003-2004.

To provide a broad assessment of the Danish U-turn, we put these Danish results in context by comparing them to the employment and earnings of non-Western immigrants in Norway and Sweden. In these analyses, Norway and Sweden can be said to act as control groups, with Denmark being the treatment group.

The results are presented as graphs illustrating the evolution of employment and earnings gaps, graphs that allow for the comparison of trends before as well as after the reforms. In the case of the crude gaps, this is simply the percentage difference between the employment ratios of natives and non-Western immigrants, and the percentage difference between the earnings of the two groups at selected percentiles of their earnings distributions. In the results based on the regression analyses, the gaps are the percentage difference in the predicted employment ratios and in predicted earnings at selected percentiles. Since we are interested in changes and not in levels, we have in all cases indexed the gaps with the gap from 1998, the year prior to the first Danish reform, set equal to 100.

While the content of the data from the three countries generally is very comparable, the extent of the information available on immigrant education and year of immigration differs. As seen in Table 1, data on level of education and on year of migration is missing in Denmark and Sweden for a sizeable proportion of non-Western immigrants, particularly at the beginning of our period. This is not an issue in Norway, and the problem also diminishes dramatically over time in Denmark and Sweden. We handle this problem by including a dummy for missing data on these two variables in all regression analyses.

**Table 1:** Descriptive statistics, selected variables

Natives	Denmark				Norway				Sweden			
	1993		2006		1993		2006		1993		2006	
	Mean	SD	Mean	SD	Mean	SD	Mean	SD	Mean	SD	Mean	SD
Woman	0.49	0.50	0.50	0.50	0.47	0.50	0.48	0.50	0.49	0.50	0.48	0.50
Child, age 0–17	0.45	0.50	0.46	0.50	0.53	0.50	0.52	0.50	0.48	0.50	0.49	0.50
Age 30–39	0.35	0.48	0.33	0.47	0.39	0.49	0.35	0.48	0.34	0.47	0.34	0.47
Age 40–49	0.37	0.48	0.35	0.48	0.38	0.49	0.34	0.47	0.38	0.49	0.34	0.47
Age 50–59	0.28	0.45	0.33	0.47	0.23	0.42	0.31	0.46	0.28	0.45	0.32	0.47
Education, comp.	0.35	0.48	0.23	0.42	0.27	0.44	0.18	0.38	0.25	0.43	0.17	0.37
Education, upp. sec. academic	0.03	0.18	0.05	0.22	0.12	0.32	0.11	0.31	0.13	0.33	0.13	0.34
Education, upp. sec. vocational	0.38	0.48	0.39	0.49	0.34	0.47	0.33	0.47	0.33	0.47	0.32	0.47
Education, tertiary	0.23	0.42	0.32	0.46	0.28	0.45	0.39	0.49	0.29	0.45	0.38	0.49
Education, missing	0.01	0.12	0.01	0.10	–	–	–	–	0.00	0.04	0.00	0.02
*Non-Western immigrants*												
Woman	0.45	0.50	0.51	0.50	0.38	0.49	0.45	0.50	0.45	0.50	0.49	0.50
Child, age 0–17	0.60	0.49	0.59	0.49	0.60	0.49	0.61	0.49	0.57	0.50	0.57	0.50
Age 30–39	0.53	0.50	0.43	0.50	0.58	0.49	0.49	0.50	0.46	0.50	0.41	0.49
Age 40–49	0.31	0.46	0.37	0.48	0.31	0.46	0.37	0.48	0.36	0.48	0.38	0.48
Age 50–59	0.16	0.36	0.20	0.40	0.11	0.31	0.15	0.36	0.18	0.39	0.22	0.41
Education, comp.	0.25	0.43	0.26	0.44	0.30	0.46	0.25	0.43	0.25	0.43	0.27	0.44
Education, upp. sec. academic	0.07	0.26	0.10	0.30	0.12	0.32	0.08	0.27	0.09	0.29	0.05	0.22
Education, upp. sec. vocational	0.20	0.40	0.25	0.44	0.14	0.34	0.11	0.32	0.30	0.46	0.28	0.45
Education, tertiary	0.20	0.40	0.23	0.42	0.44	0.50	0.54	0.50	0.26	0.44	0.37	0.48
Education, missing	0.27	0.44	0.15	0.36	–	–	–	–	0.09	0.29	0.03	0.16
Years since migr. = 0	0.04	0.19	0.03	0.16	0.02	0.14	0.06	0.24	0.01	0.08	0.02	0.14
Years since migr. = 1	0.05	0.21	0.02	0.14	0.03	0.16	0.05	0.22	0.02	0.13	0.02	0.14
Years since migr. = 2–4	0.14	0.35	0.07	0.25	0.13	0.34	0.14	0.35	0.12	0.32	0.07	0.25
Years since migr. = 5–9	0.28	0.45	0.19	0.39	0.35	0.48	0.21	0.41	0.22	0.41	0.13	0.34
Years since migr. = 10–14	0.12	0.33	0.22	0.42	0.18	0.38	0.16	0.37	0.17	0.38	0.23	0.42
Years since migr. > = 15	0.16	0.37	0.41	0.49	0.29	0.46	0.37	0.48	0.33	0.47	0.47	0.50
Years since migr., missing	0.21	0.41	0.06	0.24	–	–	–	–	0.15	0.35	0.05	0.22

Table 1 also illustrates some of the changes occurring within the immigrant populations. Noteworthy are, for instance, the increases over time in the proportion of women and older immigrants in all three countries. The increase in the female share of the Danish immigrant population may appear surprising given that family unification dropped, yet this illustrates that changes in the stock occurred at a measured pace and that earlier immigration was dominated by males. There is also a tendency towards increasing educational attainment, partly presumably due to improved educational registration. Finally, an increasing proportion of immigrants had resided in the three countries a decade or more, a result of the large number of immigrants arriving in the mid-1990s.

## Crude Employment and Earnings Gaps

**Fig. 1:**
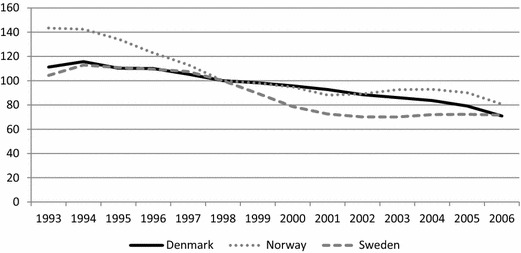
Crude employment gap between natives and non-Western immigrants in Denmark, Norway and Sweden, 1993–2006 (1998 = 100)

Figure 1 presents the development of the crude employment gaps between natives and non-Western immigrants in Scandinavia over the period 1993 to 2006, showing clear similarities in the evolution of Scandinavian immigrant labour market integration. In all three countries, the employment gap decreased more or less continuously throughout the entire 14-year period, albeit with some temporal variation. The Danish gap displays a steady decline, whereas the Norwegian drops notably in the beginning levelling off somewhat thereafter, and the Swedish gap falls in the middle of the period while staying fairly stable before and after. The narrowing of the gaps is, in all three countries, almost entirely due to rising immigrant employment; native employment rates in the three countries remained relatively stable throughout the whole period (not shown).

**Fig. 2:**
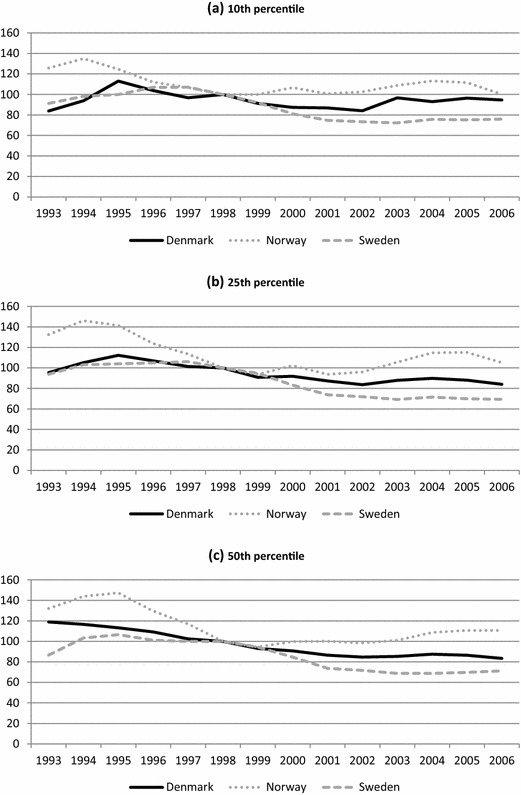
Crude earnings gap between natives and non-Western immigrants in Denmark, Norway and Sweden 1993–2006, by selected percentiles (1998 = 100). **a** 10th percentile, **b** 25th percentile, **c** 50th percentile

Turning to earnings, Fig. 2 shows the evolution of the crude earnings gap between natives and non-Western immigrants from 1993 to 2006 by selected percentiles. Here too there are signs of increasing integration, as the earnings gaps generally decrease over the observation period. The extent to which this occurs varies, however, across the earnings distribution, and across countries and over time. In Denmark, the earnings gap among the least well off (at the 10th percentile) stays relatively constant, while we see clearer evidence of continuous reductions among low (25th percentile) and median earners (50th percentile). Norway displays reduced gaps at all three points of the distribution, albeit with relatively large fluctuations over time. Sweden shows perhaps the clearest reduction in the gaps, most of it occurring in the middle of the period. It is also noteworthy that the evolution of the earnings gaps is often very similar around the time of the first Danish reform in 1998.

With regard to the Danish turnaround, the results are mixed. There is thus no indication of a marked reduction in the employment gap after the reforms in the early 2000s (or after 1998 for that matter); instead the improvement proceeds at a steady pace. Nor is there any unequivocal sign that the Danish gap improves more than the Norwegian or the Swedish, although it does continue to decline when the others remain stable towards the end of the period. Nor would the results for earnings seem to indicate any unequivocal earnings effects of the Danish reforms. At all three percentiles, the development around 1998 is characterised by a steady decline initiated some years earlier, and although the gap at the two lowest points of the distribution increases after 2002, this is later reversed. Moreover, the Swedish earnings gaps seem to have dropped more than the Danish ones throughout the lower part of the earnings distribution.

However, the immigrants’ employment rates and earnings may be affected by differences in group composition and in the business cycle, and the crude comparisons do not distinguish between immigrants affected by the various reforms and others. In the next two sections, we therefore focus on the development in predicted employment and earnings gaps for different cohorts of recent immigrants.

## Predicted Employment Gaps

**Fig. 3:**
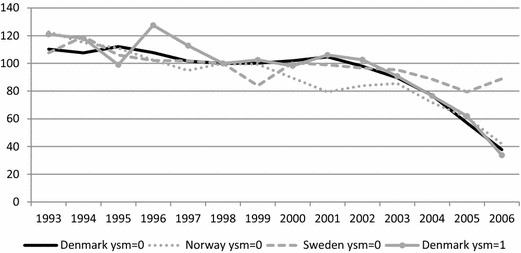
Predicted employment gap between natives and recent non-Western immigrants in Denmark, Norway and Sweden, 1993–2006 (1998 = 100). Notes: Gaps based on annual probit models including the following variables: sex, children 0–17, age 40–49, age 50–59, five educational dummies (incl. missing), and for immigrants 6 years since migration dummies (incl. missing). Predictions pertain to men aged 30–39, with a compulsory degree, at least one child aged 0–17 and living in a municipality with a 5% unemployment rate. Immigrants have furthermore lived in the destination country less than 1 year (ysm = 0) or at least one but less than 2 years (ysm = 1)

Figure 3 shows the development of the predicted employment rates for natives and non-Western immigrants from 1993 to 2006 for men aged 30–39 who had at least one child, lived in a municipality with a 5% unemployment rate, had compulsory school as their highest completed education, and had less than 1 year since migration (ysm = 0). The group has been constructed to match a presumed target group for the reforms: young recent immigrants from non-Western countries with families and a relatively low level of education. The figure also includes the predicted employment rates among a similar group of immigrants in Denmark, but with at least one and less than 2 years of residency (ysm = 1).

These estimates broadly reflect the developments already observed for the crude employment rates: a general decline in the employment gap throughout the period. This applies to all three countries, but to varying extent. The improvement in relative employment among immigrants in Denmark and Norway is thus noticeably stronger than among immigrants in Sweden. As was the case above, the declining gaps are primarily due to increasing immigrant employment, although in Denmark and Norway there is a slight decline in native employment as well (results not shown).^6^

Closer inspection of Fig. 3 reveals that in Norway and Sweden the decline in the gap is the result of a relatively steady downward trend over most of the period. The Danish development between 1996 and 2000 is roughly similar, and the similarities with Sweden continue up until 2002. Thereafter, the Danish figures deviate noticeably, showing a dramatic drop in the employment gap. The Danish decrease is furthermore quite substantial as the gap narrows to roughly a third of its previous size. The outcome of these different trends is that the Danish gap at the end of the period is practically identical to the Norwegian, and substantially smaller than the Swedish.

This drop in the predicted employment gap for recent non-Western immigrants in Denmark in the 2000s of course coincides with some of the Danish policy reforms. Thus, one interpretation of these results is that the Danish reforms in the 2000s led to increased employment rates among quite diverse groups of recent non-Western immigrants—a category that has great difficulty obtaining employment in the Scandinavian labour markets.

Before we settle on this conclusion, it is, however, interesting to look at the comparison across Danish immigrant cohorts that is between immigrants with less than one (ysm = 0) and with at least one but less than 2 years since migration (ysm = 1). Recall that the introduction of the different reforms implies that the two groups were affected by the reforms to varying extents at different points in time. It is therefore striking that the year-by-year changes in the employment gaps for the two groups are all but identical. Despite the fact that the extent to which they were affected by the reforms varied, no differences in the changes in the employment gaps are discernible. This is underscored when the standard errors of the estimates are taken into account; none of the annual estimates for the two groups of immigrants to Denmark thus differ at the 5% significance level (not shown).

This strongly suggests that the improvement in the employment gaps among these two groups was not driven by the Danish reversal of immigration and integration policy. Despite the fact that the gap drops markedly among the most recent immigrants after the reforms in 2002, and continues to do so in 2003, the same development is evident among earlier arrivals (including immigrants with ysm = 2–4, not shown) that were not (or were but to a much lesser extent) affected by the turnaround.

## Predicted Earnings Gap

The other dimension of immigrant labour market integration is earnings. We use separate quantile regression estimates for each year from 1993 to 2006 to analyse the extent to which the native-immigrant earnings gap varies across the earnings distribution when controlling for individual characteristics and unemployment rates, and in addition to the factors included in the employment regressions we here also include the sector of employment. We again focus on recently arrived young men with families and little education, comparing across countries of destination, countries of origin, men and women, and year of arrival.

**Fig. 4:**
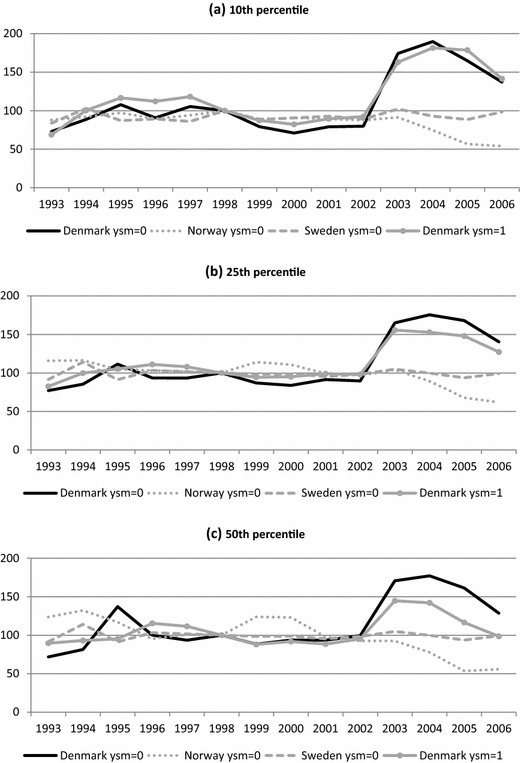
Predicted earnings gap between natives and recent non-Western immigrants in Denmark, Norway and Sweden 1993–2006, by selected percentiles (1998 = 100). **a** 10th percentile, **b** 25th percentile, **c** 50th percentile. Notes: Gaps based on annual quantile regression models including the following variables: sex, children 0–17, age 40–49, age 50–59, five educational dummies (incl. missing), ten industry dummies (incl. missing), and for immigrants 6 year of immigration dummies (incl. missing). Predictions pertain to men aged 30–39, with a compulsory degree, at least one child aged 0–17, working in the sectors wholesale and retail trade or hotels and restaurants, and living in a municipality with a 5% unemployment rate. Immigrants have furthermore lived in the destination country less than 1 year (ysm = 0) or at least one but less than 2 years (ysm = 1)

Figure 4 shows the development in earnings gap among very poor (10th percentile), poor (25th percentile) and median earners (1998 is again the base year). As is evident from the figure, the earnings gaps in Sweden remained constant throughout the whole period. The Norwegian gaps were also very stable up until 2003, after which they dropped noticeably.

The evolution of the Danish earnings gaps is quite different. Up until 2002, the gaps among the newly arrived (ysm = 0) roughly follow the Norwegian and Swedish trends. However, after 2002, the Danish gap increases dramatically at all three points of the distribution. The gaps increase further in 2003, before they start declining. Despite the drop towards the end of the period, in 2006 they remain above their pre-2002 value.

As noted, no similar deterioration is evident in the relative position of non-Western immigrants in Norway and Sweden; although they clearly are a disadvantaged group in both countries, the earnings gap remains constant or declines.^7^ This increase in the earnings gap would seem to suggest that the Danish reforms have led to a higher level of immigrant employment in low-paid work, or alternatively increased working hours in low-paid jobs.

Nonetheless, the changes in the earnings gaps among earlier arrivals to Denmark (ysm = 1) belie this interpretation, as we see a distinct increase in the gap following the 2002 reforms among this group as well. The development is basically identical across the distribution: a marked increase in 2003 followed by a slow decline until 2006. Among the very poor (10th percentile), however, the rise continues until 2005 before falling. The simultaneous rise in the earnings gaps among immigrants with ysm = 0 and ysm = 1 in 2003 are not significantly different from each other (at the 5%-level) (results not shown). Although the differences in the gaps at the 25th and 50th percentiles in 2004 are significantly (at the 5% level) greater among immigrants with ysm = 0 than with ysm = 1 (results not shown), this should not be taken as a reform effect, as by then they are affected equally by the reforms.

The strong similarity in the development of the earnings gaps for the two groups (and also broadly similar developments among immigrants with ysm = 2–4, not shown) indicates that the changes were driven by factors unrelated to the reforms. This interpretation is also strengthened by the fact that despite the increases in the gaps at the 10th and 25th percentiles, there also were reductions in earnings among natives in the period after 2002. Finally, the fact that the increases in the gaps were temporary, despite the continuation of the programmes, also suggests that it was other factors that led to the rise in inequality. While an examination of potential explanations lies beyond the scope of these analyses, the similarities in the developments suggest that the changes were caused by factors temporarily affecting the entire labour market, or at least its more precarious parts.

## Conclusions

Around the turn of the millennium the Scandinavian countries Denmark, Norway and Sweden departed from their tradition of comparable immigration and integration policies. A changing mixture of rights and duties developed, with Denmark restricting immigration and tightening labour market integration policies in ways not seen in Norway and Sweden. The impact of the Danish reforms could be expected to be varied. It is for instance possible that the reforms could to lead to higher employment rates among immigrants, yet if this is the result of more immigrants taking up low-paying jobs, there could also be a concomitant increase in the earnings gap.

To understand the impact of the Danish policy reversal, we have carried out multiple comparisons: between countries of destination, across countries of origin and among immigrant cohorts. We have examined both crude employment and earnings gaps as well as differentials based on estimates from regression models. Our analyses suggest three broad conclusions. First, there was a general improvement in the labour market integration of immigrants to Scandinavia during the years 1993–2006. Employment and earnings gaps declined, both in the population as a whole and among specific groups. These improvements were not equal or uninterrupted, being more evident with regard to employment and to earnings among median earners, for example, than to earnings among the less well off. Nonetheless, these two central measures of immigrant integration generally improved.

Second, the Danish reforms to immigration and labour market integration policy seem to have had a very limited impact on immigrant integration in Denmark. While we find that both employment and earnings gaps change following the reforms in ways predicted by theory, similar changes occurred among immigrant groups affected by the reforms and among those less impacted by them. This indicates that other factors, factors outside of our analysis, played a greater role in the evolution of immigrant integration on the Danish labour market.

Third, the importance of factors other than immigration and integration policies is underscored by the fact that immigrant labour market integration often, but not always, improved as much in Norway and Sweden as in Denmark. Crude employment gaps dropped as much in Sweden, while crude earnings gaps dropped more. Predicted employment gaps improved as much in Norway, while predicted earnings gaps improved more. The lack of an unequivocal reform effect in Denmark does clearly not absolve policy makers from considering reforms to measures targeted at immigrants. Nonetheless, the roughly similar improvement in the labour market integration of immigrants in Norway and Sweden suggests that it may be necessary to think beyond the Danish policies and consider other measures as well.

These last points would seem particularly relevant in the light of more recent Scandinavian developments. The Danish Start Help programme as well as the social assistance ceiling was abolished in 2012, yet the programme was reintroduced in 2015 and set at approximately 45% below the level of social assistance. Moreover, a 24-year requirement very similar to the Danish one was introduced in Norway in 2013. While one should be wary of extrapolating both from one programme to another and across settings, based on our analysis of the Danish reforms around the turn of the millennium these new initiatives would seem unlikely to markedly improve the labour market integration of immigrants.

Caution is of course equally warranted with regard to the implication of the results for other countries. Nonetheless, it seems safe to say that stricter immigration and labour market integration policies do not automatically lead to greater labour market integration, nor do such policies seem to be the only way to achieve such a goal.

## Footnotes

1 This presentation of Scandinavian policy development focuses on the period leading up to and including our analyses, and therefore ends in approximately 2010. Subsequent reforms are briefly discussed in the concluding section.

2 While the means-tested character of social assistance benefits makes international comparison problematic, analyses of the Social Assistance and Minimum Income Protection data set (Nelson 2007) indicate that social assistance “on average” was most generous in Norway and least so in Sweden. The data set contains no information on immigrant benefits specifically, yet after the cutbacks, the benefits available to immigrants in Denmark would seem to have been lower than in the other countries.

3 Search theory also generates hypotheses regarding other aspects of the benefits and programmes that may partly contradict those outlined above, e.g. lock-in and threat effects. However, these would appear to be of secondary importance relative to the ones above.

4 One drawback of the data is that we have been unable to pool the data from the three countries for reasons of confidentiality.

5 Focusing on a single reform would, for example, allow us to make use of a regression discontinuity design, yet our interest in the broad impact of the Danish turnaround precludes this.

6 The development of predicted employment gaps for recent immigrants from Turkey, the Horn of Africa and Poland (not shown) are broadly in line with the results for the group of non-Western immigrants as a whole, although the reduction in the employment gap in all three Scandinavian countries is greatest among immigrants from Poland followed by immigrants from Turkey. Likewise, separate analyses of the employment gaps among men and women (not shown) reveal these too to be in general agreement with our overall results, although the reduction in the employment gap is somewhat smaller among women than among men.

7 The analyses of immigrants from Turkey, the Horn of Africa and Poland again indicate that the developments among these three groups are broadly in line with the general development within each country (not shown). One exception is, however, Polish immigrants in Sweden for whom there is a reduction in the earnings gap at all three points of the distribution. The developments among men and women are also in general agreement with the overall pattern in each country (not shown).
